# Targeting Malignant Brain Tumors with Antibodies

**DOI:** 10.3389/fimmu.2017.01181

**Published:** 2017-09-25

**Authors:** Rok Razpotnik, Neža Novak, Vladka Čurin Šerbec, Uros Rajcevic

**Affiliations:** ^1^Department of Research and Development, Blood Transfusion Centre of Slovenia, Ljubljana, Slovenia

**Keywords:** antibody, glioma, bispecific Ab, blood–brain barrier, receptor-mediated transcytosis, cell-penetrating peptides, single-chain fragment variable, chimeric antigen receptor-T cell

## Abstract

Antibodies have been shown to be a potent therapeutic tool. However, their use for targeting brain diseases, including neurodegenerative diseases and brain cancers, has been limited, particularly because the blood–brain barrier (BBB) makes brain tissue hard to access by conventional antibody-targeting strategies. In this review, we summarize new antibody therapeutic approaches to target brain tumors, especially malignant gliomas, as well as their potential drawbacks. Many different brain delivery platforms for antibodies have been studied such as liposomes, nanoparticle-based systems, cell-penetrating peptides (CPPs), and cell-based approaches. We have already shown the successful delivery of single-chain fragment variable (scFv) with CPP as a linker between two variable domains in the brain. Antibodies normally face poor penetration through the BBB, with some variants sufficiently passing the barrier on their own. A “Trojan horse” method allows passage of biomolecules, such as antibodies, through the BBB by receptor-mediated transcytosis (RMT). Such examples of therapeutic antibodies are the bispecific antibodies where one binding specificity recognizes and binds a BBB receptor, enabling RMT and where a second binding specificity recognizes an antigen as a therapeutic target. On the other hand, cell-based systems such as stem cells (SCs) are a promising delivery system because of their tumor tropism and ability to cross the BBB. Genetically engineered SCs can be used in gene therapy, where they express anti-tumor drugs, including antibodies. Different types and sources of SCs have been studied for the delivery of therapeutics to the brain; both mesenchymal stem cells (MSCs) and neural stem cells (NSCs) show great potential. Following the success in treatment of leukemias and lymphomas, the adoptive T-cell therapies, especially the chimeric antigen receptor-T cells (CAR-Ts), are making their way into glioma treatment as another type of cell-based therapy using the antibody to bind to the specific target(s). Finally, the current clinical trials are reviewed, showing the most recent progress of attractive approaches to deliver therapeutic antibodies across the BBB aiming at the specific antigen.

## Introduction

Approximately 27,000 new cases of malignant glial tumors are diagnosed in Europe every year. The most common are glioblastoma multiforme (50%) and anaplastic glioma (10%) (1). They are associated with high morbidity and mortality because they are highly invasive and neurologically destructive (2). Gliomas penetrate throughout the brain and extend far beyond the tumor mass that is visible with neuroimaging, making them difficult to treat (3). Despite surgical resection, radiotherapy, and chemotherapy, the median survival time is only 14–15 months for patients with glioblastoma (4) and 2–5 years for those with anaplastic gliomas (2). New approaches to treatment are needed to improve the prognosis. A promising one is antibody (Ab; in this review, the acronym Ab is used for all forms of antibodies and their fragments, unless stated otherwise) therapy, which is discussed in this review.

Targeting brain diseases such as brain cancer and neurodegenerative diseases with therapeutics is especially challenging because of the presence of the blood–brain barrier (BBB). BBB has an extremely low permeability, which helps to maintain brain homeostasis (5). In the case of brain tumors, the BBB faces some abnormalities where, besides the morphological changes in the barrier, its permeability increases because of disrupted junctions in the layer of endothelial cells. However, increased permeability during some pathological processes still does not suffice for the passage of larger molecules such as biologicals. Crossing the BBB would facilitate the Abs to reach their targets and execute their therapeutic potential. The permeability of BBB can be achieved through invasive and non-invasive methods. Invasive methods (e.g., focused ultrasound, osmotic disruption, biochemical disruption) pose certain risks of infections, toxicity, and damage to the brain. Non-invasive methods represent a much safer and convenient way for the delivery of therapeutics (6).

This review will focus on antibody tools for the treatment of malignant gliomas with different mechanisms of passage through the BBB. Several approaches, including cell-based approaches, will be discussed with their future potential, and the currently active clinical trials will be overviewed.

## Crossing the BBB

Transcellular mechanisms of transport such as adsorption-mediated transcytosis (AMT), and particularly receptor-mediated transcytosis (RMT), have gained most interest and have shown the highest potential for the non-invasive delivery of therapeutics through the BBB into the brain (5). In AMT, positively charged molecules can interact with the negatively charged membrane of endothelial cells, upon which endocytosis and crossing of the BBB can occur. The entire process is receptor independent and non-specific (5). Several mechanisms of AMT are being explored with potential therapeutic Abs (7–9). Cationized F(ab′)_2_ fragment against Aβ plaques have shown increased permeability across the BBB (10). Other cationized proteins that could serve as carrier proteins were also investigated-for example, cationized protein G for the delivery of IgG antibodies (11). AMT is also being investigated as a mechanism for the passage of nanoparticles where targeting brain tumors with cationized liposomes has shown great promise. Cationization has not only provided an efficient passage through BBB but has also served to enhance the binding of nanoparticles to the tumor endothelium (12–14).

Binding to specific receptors has promoted the transcytosis of a bound ligand where dissociation of the bound complex occurs after being transported across the cytoplasm. Certain peptides or proteins such as insulin and transferrin enter the brain tissue by RMT where they bind to a specific receptor expressed on the luminal side of the BBB. Some of the most studied receptors for targeting brain tissue and promoting passage through the BBB are the insulin receptor (InsR), LDL-related protein type 1 (LRP1) Receptor and transferrin receptor (TfR) (15, 16). Another way to mediate RMT is to target specific receptors using Abs that recognize and bind to them, a strategy known as the “Trojan horse” method. Therapeutics can be designed as bispecific Abs (bsAbs) where one Ab has specificity toward a receptor expressed on the luminal side of the BBB and the other has specificity toward a therapeutic target (17). Therapeutics can also act as a cargo where they are conjugated to a receptor targeting Abs. Another interesting strategy is to use cell-penetrating peptides (CPP) as a Trojan horse for the delivery of therapeutics to brain tissue (18). All these strategies will be discussed further on in later sections.

## Receptors Mediating RMT

The most common receptors for mediating RMT (TfR, InsR, LRP1 receptor) have been successfully used for passing the BBB (19). However, they have all shown potential drawbacks. Their expression profile is not specific for brain tissue (20–22), causing side effects (acute clinical signs and decreased reticulocyte count) (23). The drawbacks of existing model receptors for passing the BBB (19, 23, 24) have led scientists to identify new potential target receptors in the BBB (24). Since abnormalities occur in the BBB in brain tumors, the expression of potential receptors that mediate RMT must be investigated specifically for the blood–brain tumor barrier (BBTB). For instance, some membrane transporters have been found to be over-expressed in the BBTB [e.g., P-glycoprotein (P-gP), multidrug resistance-associated protein 1 (MRP 1) and 3 (MRP3)] (6).

## Ab Properties Necessary to Pass the BBB-Abs That Serve as a Trojan Horse

Nearly 50% of Abs used in malignant glioma clinical trials are intact IgG Abs (6). These conventional Abs can remain in the peripheral circulation for days to weeks. Although their persistence in the peripheral system offers a therapeutic advantage, they can exhibit poor tissue penetration due to their large size. This is especially true in the case of targeting brain tissue and crossing the BBB (25). In mouse models, it has been reported that less than 0.1% of peripherally administered Abs can reach the brain tissue, with evidence indicating that only approximately 0.009 ± 0.001% of the injected dose of systematically administered intravenous immunoglobulins reached the cortex (26). The concentration of IgG Abs in the brain is additionally rapidly decreased through the activity of a neonatal Fc receptor (FcRn) which promotes reverse transcytosis. This could also be an advantage if the mechanism of accelerated circulation of IgG with the repeated transition of IgG is favorable. However, if prolonged exposure to higher concentrations of IgG is favorable, then FcRn-mediated efflux represents a disadvantage. Several solutions have been provided to escape FcRn-mediated efflux (27). Fc inhibition (28), and the use of low-affinity FcRn activity Abs (29) have successfully reduced the efflux of Abs from brain tissue. Alternatively, the use of Ab fragments lacking the Fc region avoids this problem.

Abs must possess certain properties to play a role as a Trojan horse by mediating RMT and crossing the BBB. The binding of Abs should not interfere with the binding of endogenous proteins and should promote receptor-mediated endocytosis. Manipulation of Abs that bind TfR, by decreasing their affinity (30, 31) and shifting their valency from bivalent to monovalent (32) has been shown to increase the successful delivery of Abs. Bivalent (32) and monovalent high-affinity (31) anti-TfR Abs have been associated with lysosomal degradation due to potential dimerization of the TfR receptor (32) or have predicted poor dissociation from the Ab–receptor complex (30). It can also be speculated that different epitopes on the extracellular part of TfR play an important role, but this has yet to be evaluated.

## Alternative Forms of Ab

Other Ab formats have been investigated for the treatment of brain tumors. Smaller Ab formats such as Fab or scFv possess several advantages over the use of conventional Ab formats. scFvs have been the most studied Ab fragment format for targeting brain diseases. Their small size improves tissue penetration. They are also easier to produce and genetically modify. The lack of an Fc region offers the advantage of circumventing FcRn-dependent efflux from brain tissue and eliminates Ab effector functions, such as complement-dependent cytotoxicity (CDC) and Ab-dependent cellular cytotoxicity (ADCC) where further inflammatory stimuli are prevented (33). These two characteristics offer a special advantage regarding targeting brain tissue. However, the absence of an Fc region also shortens the Ab half-life. Several techniques, e.g., the addition of PEG and conjugation of scFv to other proteins or molecules prolong their half-life. scFvs have been used to target brain tissue in the form of bispecific T-cell engagers (BiTE) (34), conjugated to liposomes (35), and linked with CPP (18, 36). They have also served as a Trojan horse where they target TfR and successfully mediate the passage of a conventional anti-Aβ Ab (37). To our knowledge, passive passage of scFv across the BBB has not been directly compared with conventional Abs and remains to be evaluated. Several scFvs have shown therapeutic potential when targeting glioblastoma *in vivo*; however, most of them target non-orthotopic xenografts (38), or circumvented the BBB by direct distribution using convection-enhanced delivery (CED) (39, 40) and intracerebral injection (41). scFv D2C7 linked to immunotoxin targeting glioblastoma is in the phase I clinical trial stage and is being tested by intratumoral CED (42). Although CED represents a promising drug delivery method (43), it still is an invasive method.

## CPPs as Another Key Strategy to Cross the BBB

**Figure 1:**
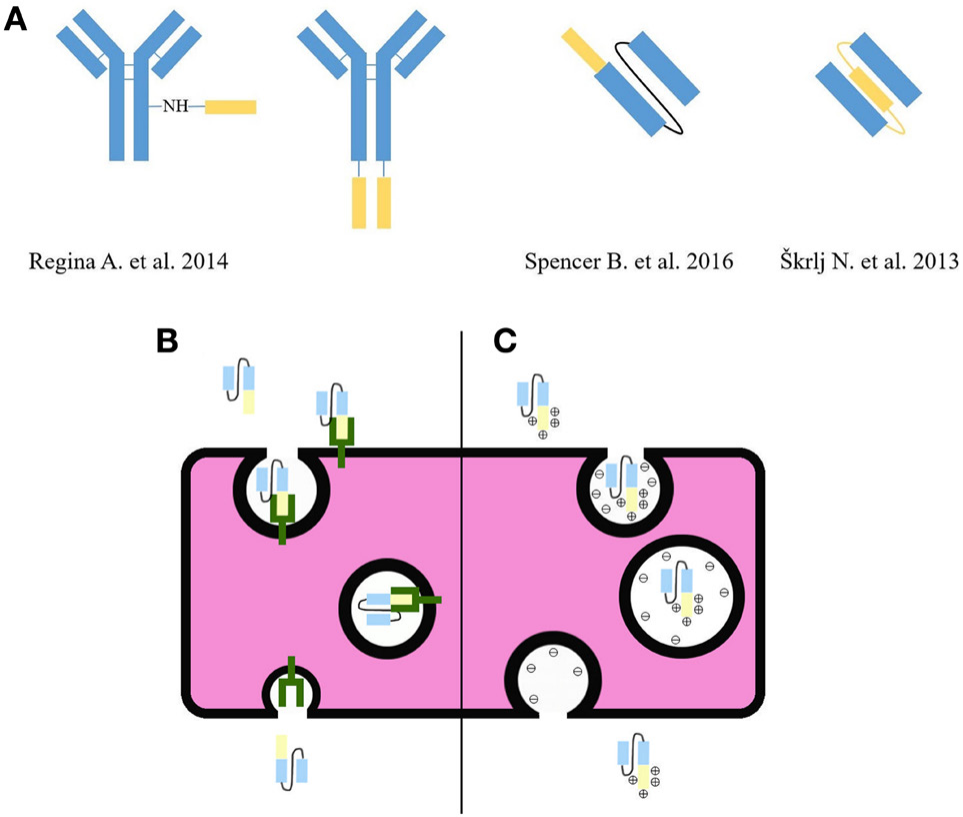
**(A)** Flexibility of cell-penetrating peptide (CPP) incorporation into the Ab scaffold. Some examples efficiently passed through the BBB (18, 36, 44). The yellow color indicates CPP and the blue color indicates Abs. **(B)** CPPs can mediate RMT by binding receptor at the BBB and transporting the Ab across the cytosol to the other side of the BBB. **(C)** CPPs consisting of amphipathic and/or cationic sequences can mediate AMT and allow crossing the BBB.

Cell-penetrating peptides are a group of short peptides, consisting of amphipathic and/or cationic sequences that enable crossing the cell membranes (Figure 1C). From a therapeutic point of view, they can be conjugated to therapeutics (e.g., Ab-based) and can be used to mediate their passage through the BBB. Generally, their uptake is non-specific without the need of a transporter. Although the mechanisms of passage are still under investigation for some CPPs, AMT is the main mechanism. The non-specific uptake of peptides can be solved by incorporating a receptor targeting scaffold, as it has been shown for bi-functional liposomes conjugated to CPPs and transferrin, with improved BBB penetration compared with liposomes without included CPPs. Improved penetration most likely occurs because the incorporation of CPP overcomes receptor saturation (16). Some CPPs have been shown to target transporters at the BBB and mediate RMT (Figure 1B) (15). Their conjugation to therapeutic Abs allows efficient delivery of Abs into the brain tissue. Anti-human epidermal growth factor receptor 2 (HER2) monoclonal Ab conjugated to Angiopep-2 peptide, which binds to the LRP1 receptor in the BBB, efficiently passed through the BBB and prolonged the survival of mice with BT-474 brain tumor xenografts after systematic treatment (44).

Besides cell-penetrating properties, some peptides such as iRGD-amino-acid sequence: C(RGDKGPDC) have also a more specific feature of tumor penetration. iRGD possesses affinity toward the tumor vasculature-specific αv integrins. After proteolytic cleavage, iRGD gains affinity toward neuropilin-1 (NRP-1) where it mediates further tumor tissue penetration (45). Nanoparticles conjugated to iRGD or co-administered with iRGD peptides have shown increased crossing of the BBB and enhanced intratumoral accumulation levels in glioma mouse models (46). Doxorubicin liposomes conjugated to a NRP-1-specific tumor-penetrating peptide prolonged the survival in mice and effectively crossed the BBB (47). Tumor-penetrating peptides represent a promising strategy for their ability to cross the BBB and specifically penetrate the tumor tissue. Inclusion of therapeutic Abs in the liposomes conjugated to tumor-penetrating peptides may provide further success in this field. Tumor-penetrating peptides have also been used successfully when linked to Abs or administered together. TPP11, an NRP-1-specific peptide that also blocks the interaction of NRP-1 with vascular endothelial growth factor (VEGF), was linked to the Fc region of an anti-epidermal growth factor receptor (EGFR) monoclonal Ab. The design has allowed good tumor penetration and accumulation and has further presented anti-angiogenesis activity (48). The anti-HER2 Ab trastuzumab, when co-administered with iRGD, completely eradicates all tumors in orthotopic BT474 human breast tumor cell xenograft mouse models, whereas treatment with trastuzumab alone slows down tumor growth (45). The conjugation of tumor-penetrating peptides to Abs or their co-administration may provide enhanced therapeutic targeting of brain tumors.

Our research group has also provided a proof of flexibility in the design of Ab fragments coupled to CPP. Normally CPPs are conjugated to the C- or N-terminal end of an Ab molecule or other chemical groups on Ab (Figure 1). Our group has successfully prepared single-chain fragment variable (scFv) against the truncated form of prion protein with a penetratin used as a linker between the two variable domains. This design has allowed the passage of scFv through BBB (18). Altogether, CPPs present a prospective method to increase the brain uptake of different therapeutic Abs. New peptides against potential receptors in the BBB can be selected by phage display biopanning (49).

## Nanoparticles and Liposomes—A Vehicle for the Delivery of Therapeutics into the Brain

Nanoparticles and liposomes have proven to be efficient tools for the delivery of Ab-based therapeutics into the brain; particularly, liposomes have been extensively used to target glioma. The passage of liposomes through the BBB has already been shown to be increased by cationization (12–14), conjugation to Abs (50, 51), CPPs (47, 52–54), protein ligands of receptors at the BBB (55) or conjugation to two of them, namely protein ligands of receptors at the BBB and CPPs (16, 56). Another strategy to increase the passage through the BBB is the use of magnetoliposomes, where magnetic nanoparticles are incorporated into liposomes and external magnetic fields are used for guidance across the BBB (57). Liposomes conjugated to Abs to cross the BBB or specifically target tumor tissue are called immunoliposomes, and they are being studied extensively for the targeted drug delivery to tumor tissue. Cationic liposomes, encapsulating temozolomide and conjugated to anti-TfR scFvs, show prolonged survival and inhibition of tumor growth in an intracranial glioblastoma xenograft (U87-luc2) model (58). Liposomes can incorporate hydrophilic, hydrophobic, and lipophilic substances due to their composition where one or more lipid bilayers surround an aqueous compartment. Therefore, they are suitable for the delivery of various drugs, including Abs (59). The administration of some therapeutic Abs may lead to off-target effects and cytotoxicity. Encapsulating those in liposomes and providing specific delivery may help to circumvent this problem (60). In addition to controlled drug release and specific delivery, liposomes also present good biocompatibility, biodegradability, and low toxicity. Although only passive targeting strategies using liposomes are currently in clinical trials, their drawbacks include poor penetration through the BBB, non-specific uptake and a low enhanced permeability and retention effect (EPR) (61). However, active targeting strategies using (tumor) penetrating peptides and Abs against receptors present in the BBB have improved their therapeutic potential. Therefore, immunoliposomes and liposomes conjugated to (tumor) penetrating peptides present an interesting, more specific targeting strategy, with controlled release of therapeutics and provide a promising strategy for targeting brain tumors. Apart from liposomes, nanoparticles can successfully cross the BBB. This can be accomplished by conjugation of nanoparticles to protein ligands of receptors at the BBB (62), CPPs (63), and Abs (64, 65). Nanoparticles also serve as carriers of various drugs, where they can be adsorbed, covalently bound or encapsulated.

## Bispecific Abs (bsAbs)—A Promising Technology

Bispecific Abs recognize two different epitopes. Many different technologies to produce bsAbs have been described (66, 67). The passage through the BBB can be mediated using a bsAb where one Ab’s specificity recognizes a receptor at the BBB, which then promotes transcytosis. The other Ab’s specificity recognizes a potential therapeutic target. Within this scaffold, a therapeutic potential and ability to promote crossing over the BBB are combined in one molecule. Until now, only bsAbs targeting TfR and beta-secretase 1 (BACE1) have been described (30, 68). The same mechanism of action could be used to target brain tumors, where one specificity would target a receptor suitable for RMT, while the other would target a tumor-specific or tumor tissue-overexpressed antigen. Affinities for specific epitopes may change when designing bsAbs; therefore, affinities for both epitopes should be adjusted to allow efficient delivery and therapeutic response.

Bispecific Abs have also been proven to mediate a more efficient therapeutic response when targeting two epitopes simultaneously. Treating (non-brain) tumors with VEGF inhibitors alone promotes tumor metastasis, VEGF-independent angiogenesis and increased hypoxia (69). Angiopoietin-2 (Ang-2), an angiogenic growth factor, was overexpressed in bevacizumab-treated glioblastomas, while translocator protein (TSPO) was upregulated in bevacizumab-treated glioblastomas and promoted apoptosis resistance. Targeting both epitopes with bsAb in bevacizumab-treated rats resulted in significantly prolonged survival and showed promise for the treatment of the aggressive and apoptotic-resistant nature of bevacizumab-treated glioblastomas (70). Another bsAb targeting Ang-2 and VEGF prolonged survival and provided other clinical benefits in a mouse brain tumor model with glioblastoma xenografts (71). However, how these bsAbs passed through the BBB has not been evaluated. One possible speculation is that small concentrations of these therapeutic Abs are sufficient for the therapeutic effect. On the other hand, Ang-2 upregulation is associated with BBB disruption and enhanced paracellular and transcellular passage (72). It could be considered that, in glioblastoma, where Ang-2 is overexpressed, the passage of Abs across BBB is enhanced through a passive mechanism.

Redirection of immune cells to target tumor cells using bsAbs offers another promising mechanism of treatment. By linking a tumor-specific epitope to a T-cell activated ligand, an immune synapse is formed. Particularly, a successful group of bsAbs in this field turned out to be a group of BiTEs, where two scFvs, each targeting its own antigen, are linked together in tandem. A BiTE, targeting a specific T-cell activated ligand, CD3, and tumor-specific mutated EGFR receptor (EGFRvIII) that is constitutively activated and is often found in glioblastoma, had promising therapeutic effects in mice using a human glioblastoma xenograft model U87MG.ΔEGFR. Treating affected mice resulted in prolonged survival, and, in the case of higher dosages, the mice were completely cured without apparent cytotoxicity (73). However, the mechanism of passage across the BBB remains unknown and was not investigated in many cases of targeted brain tumor models (Table 1). Enhanced passage was predicted due to the reduced size of BiTEs compared with conventional Abs, and it was assumed that they elicited their effect even when present in considerably low concentrations (74).

**Table 1:** Ab-based therapies targeting glioma models *in vivo*, their proposed mechanism of passage and their therapeutic outcomes (2013–present).

	Therapeutic agent	Mechanism of passage	Brain tumor model	Therapeutic outcome	Referece
1	ANG-4043: anti-HER2 Ab conjugated to CPP Angiopep-2	RMT	Intracranial breast ductal carcinoma^a^ xenograft (BT-474) in mice	Increase in median survival (for 80 days)	(44)
2	Anti-Ang-2/TSPO bispecific Ab	Unknown	Intracranial glioblastoma xenograft (GL261) in mice; glioblastoma bearing rats treated with bevacizumab prior to treatment	Reduced tumor size and increased survival in mice; increased overall survival and reduced macrophage infiltration in rats	(70)
3	Anti-Ang-2/VEGF bispecific Ab	Unknown	Intracranial glioblastoma xenografts (GL261, MGG8) in mice	Decreased vessel density, delayed tumor growth, prolonged survival, reprogramming of macrophages in GL261 mice; prolonged survival and reprogramming of macrophages in MGG8 mice	(71)
4	Anti-EGFRvIII/CD3 BiTE	Unknown	Intracranial glioblastoma xenograft (U87MG.ΔEGFR) in mice	Prolonged survival and complete cure rates up to 75%	(75)
5	NZ-1-(scdsFv)-PE38KDEL: anti-podoplanin immunotoxin	n/a—CED	Intracranial medulloblastoma^a^ (D425MED) xenograft in mice	Increase in survival (41%)	(39)
6	D2C7-(scdsFv)-PE38KDEL: anti-EGFR/EGFRvIII immunotoxin	n/a—CED	Intracranial glioblastoma xenografts (43MG, NR6M and D270MG) in mice	Increased survival (43MG by 310%, NR6M by 28%, D270MG by 160%)	(76)
7	IP10-EGFRvIII scfV	n/a—i.c.	Intracranial glioblastoma xenograft (U87MG.ΔEGFR) in mice	Reduced tumor growth and prolonged survival	(77)
8	Anti-PD-1 Ab (±radiation therapy)	Route of administration is unknown	Intracranial glioblastoma xenograft (GL261-Luc) in mice	Long-term survival (180 + days) for 15–40% of animals	(78)
9	Ficlatuzumab (±temozolomide)	Unknown	Intracranial glioblastoma xenograft (U87MG) in mice	Prolonged survival in monotherapy. More prolonged survival in combination therapy where 80% of animals remained free of clinical signs of the disease after treatment	(79)
10	mAb9.2.27: anti-NG2 Ab (±NK cells)	n/a—intra-lesional treatment	Intracranial glioblastoma xenografts (U251-NG2, U87MG) in rats	Prolonged median survival time (combination therapy: U251-NG2 for 5,5 days and U87MG for 52 days)	(80)
11	AMG 595: Ab drug conjugate anti-EGFRvIII conjugated to DM1	Unknown	Intracranial glioblastoma xenograft [D317(EGFRvIII positive)] in mice	Inhibition of tumor growth	(81)
12	TTAC-0001: anti-VEGFR-2/KDR Ab	Unknown	Intracranial glioblastoma xenograft (U87MG) in mice	Inhibition of tumor growth	(35)
13	Nanocomplex scL-TMZ: cationic liposomes encapsulating temozolomide and conjugated to anti-TfR scFv	RMT	Intracranial glioblastoma xenograft (U87-luc2) in mice	Inhibition of tumor growth, prolonged survival	(58)
14	Anti-EGFRvIII Ab + rapamycin	Unknown	Intracranial glioblastoma xenograft (U251-EGFRvIII) in mice	Prolonged median survival time (combination therapy by 31,5 days)	(82)
15	Anti-Ang2 Ab + cediranib	Unknown	Intracranial glioblastoma xenografts (U87, GL261) in mice	Prolonged median survival time (combination therapy U87 by 21 days and GL261 by 18 days), slower tumor growth rate in the GL261 model, development of early necrosis in the U87 model, structural vessel normalization in both models, alteration of tumor-associated macrophages	(83)
16	Anti-CD47 Ab	Unknown	Intracranial glioblastoma xenografts (GBM4, GBM5) in mice	Reduced tumor burden, survival benefit, alteration of tumor-associated macrophages	(84)
17	Anti-GITR Ab + radiation therapy	Unknown	Intracranial glioblastoma xenograft (GL261-luc) in mice	Combination therapy: improved survival, delayed tumor progression, a subset of cured long-term survivors	(85)
18	Anti-CD40 Ab	n/a—CED	Intracranial glioblastoma xenografts (GL261, NSCL61, bRiTs-G3) in mice	Prolonged survival	(86)
19	Bevacizumab	n/a—transcranial focused ultrasound	Intracranial glioblastoma xenograft (U87) in mice	Increase in median survival time (135%)	(87)

*^a^Not glioma models*.

## Stem Cells (SCs) as Delivery Vehicles for Ab to Tumors

Stem cells are a promising strategy for *in vivo* Ab production and delivery, mainly because of their pathotropism properties and ability to cross the BBB (88, 89). Mesenchymal stem/stromal cells (MSCs) are multipotent and can differentiate into many adult cell types of mesenchymal origin (90, 91). Neural stem cells (NSCs) have self-renewal capacity and multipotent potential to differentiate into neurons, astrocytes, and oligodendrocytes (92–94).

The major problem of treating malignant gliomas is that they infiltrate the surrounding normal brain tissue and are elusive to standard therapies. MSCs and NSCs from different sources have significant tropism to tumors and are usually used in studies of therapeutic protein delivery. It was shown that both NSCs and MSCs have tumor tropism properties and can migrate toward malignant glioma, distribute across the tumor bed and continue expressing a foreign gene (95–97). NSCs were observed while migrating from the transplantation site to the tumor. They were clearly tumor tropic, but some migrated to other areas such as the hippocampus and auditory cortex (98).

Understanding the mechanisms regulating SC migration is necessary to optimize the use of SCs as therapeutic delivery vehicles (99). Glioma cells produce their own extracellular matrix (ECM) and invade the surrounding brain parenchyma by expression of additional ECM molecules, including tenascin, fibronectin, laminin, vitronectin, and different types of collagen (100). The ECM of malignant glioma facilitates NSC migration *in vitro*. When different ECM molecules were tested for NSC migration, laminin was the most permissive, whereas tenascin, fibronectin, and vitronectin also supported NSC motility (101).

It was shown that NSCs preferentially target hypoxic glioma regions *in vivo*. Knockdown of HIF-1α, which is a master regulator of many genes involved in tumors, resulted in the inhibition of hypoxia-induced NSC tropism. Hypoxia is a key factor for NSC tropism and the process is mediated by stromal derived factor 1/chemokine receptor type 4 (SDF-1/CXCR4), urokinase-type plasminogen activator/its receptor (uPA/uPAR), VEGF/VEGFR2, and hepatocyte growth factor (HGF)/c-Met signaling pathways (102). HGF and other growth factors [VEGF, epidermal growth factor (EGF) and transforming growth factor α (TGFα)] can also induce the migration of NSCs. This is similar to the migration of cancer cells in glioma invasion, only that it is deregulated and constitutive (103). IL-8 appears to be another chemoattractant promoting SC migration. The migration of MSCs toward a glioma cell line was enhanced also by the overexpression of its receptor chemokine receptor 1 (CXCR1) in MSCs. This implies that the overexpression of CXCR1 could be a way of improving MSC tropism in glioma therapy (104). It was also shown that both MSCs and NSCs show significantly greater migration toward cancer cell lines of solid tumors that express high levels of uPA and uPAR compared with those with low uPA/uPAR expression. Therefore, MSCs and NSCs can use multiple cytokines for tropism to tumors, but a common feature is the expression of uPA and uPAR (105).

The migratory capacities of MSCs and NSCs to brainstem glioma were compared *in vitro* and *in vivo*, and it was shown that MSCs from various sources have similar migratory capacities to NSCs. It was also reported that not all but only approximately 30% of all SCs migrated to the target glioma from the injection site (forebrain). It is possible that only astrocytic precursors migrate to the tumor (106). Understanding the mechanism of NSC glioma targeting can help in designing genetically engineered NSCs with optimal cytokines and receptor combination for effective NSC migration and drug delivery to solid tumors.

The tumor tropism of SCs can be exploited to deliver therapeutic agents selectively to tumors. MSCs were first tested for the delivery of therapeutic proteins to tumors in pulmonary metastases (97) and later on gliomas using an intracranial glioma model and hMSCs engineered to release interferon beta (IFN-β) (99). For NSCs, it was reported that, using an immortalized NSC cell line expressing an anti-cancer prodrug (rCE; activates CPT-11), a tumor-free survival of 100% of mice (model of pediatric neuroblastoma) for longer than 6 months was achieved. MSCs continue to replicate *in vivo* and incorporate into tumor stroma and could possibly support tumor growth. They also engraft in the bone marrow of recipients, whereas NSCs are only detectable in the bone marrow if tumor cells are present. Thus, it was proposed that NSCs may be preferable to MSCs when a relatively short-term survival of SCs is desirable, such as in cancer therapy (107). These pioneer studies serve as a foundation for other SC therapies combined with Abs against glioma or other cancers. Studies where SCs expressing Abs were used are summarized in Table 2 and are described below.

**Table 2:** Therapies with SCs expressing Abs and Ab fragments against brain tumor antigens and their outcome in preclinical studies.

	Stem cell	Therapeutic protein	Brain tumor model	Outcome	Reference
1	NSC HB1.F3	Full length anti-HER2 Ab (trastuzumab equivalent)	Breast cancer brain metastases (BT474Br cells)	Significant improvement of survival in mice (approximately 30 days)	(108)
2	NSC	EGFR-specific nanobodies (ENbs) and ENb2-TRAIL immunoconjugate	Intracranial glioblastoma model (U87)	Significant inhibition of tumor growth with NSC-ENb2 and complete prevention of outgrowth with NSC-ENb2-TRAIL; increased survival; inhibition of tumor invasiveness	(109)
3	hMSC	Anti-EGFRvIII scFv	Intracranial glioma xenografts (U87-EGFRvIII)	Survival prolonged for 1 week in mice; an additional injection further prolonged survival	(110)

Neural stem cells were genetically engineered to secrete properly assembled anti-HER2 Ab (trastuzumab equivalent), which can inhibit the proliferation of HER2-positive breast cancer *in vitro*. GM NSCs could deliver these Abs to human breast cancer xenografts in mice. The anti-HER2 Ab was detected only at the tumor site but not in the blood of NSC-treated mice, showing the potential for a robust localized anti-tumor effect with minimal systemic toxicity (111). In a later study, the anti-HER2 Ab SC therapy was tested for its efficacy against brain tumors (Table 2). In a breast cancer brain metastases mouse model, the intracranial injection of NSCs secreting anti-HER2 Ab showed a significant improvement in survival. It was reported that anti-HER2 Ab secreted by NSCs binds to HER2-overexpressing human breast cancer cells and inhibits PI3K–Akt signaling and inhibits growth *in vitro*. PI3K–Akt signaling is activated by HER2 dimerization and leads to increased invasion responsible for metastatic breast cancer. These benefits are not efficient against brain metastases if the Ab fails to penetrate the BBB (108).

Neural stem cells were also tested for the delivery of EGFR-targeting nanobodies (ENbs) or ENb-derived immunoconjugates (Table 2). They maintained transgene expression *in vivo* and *in vitro* over a period while maintaining stem properties. ENbs secreted by NSCs inhibited EGFR signaling *in vitro* and reduced glioblastoma growth in mice but did not result in significant regression of the tumor size. To increase the efficacy, an ENb2—tumor necrosis factor-related apoptosis-inducing ligand (TRAIL) immunoconjugate was designed. This induced caspase-3/7-mediated apoptosis in GBM cell lines with various degrees of TRAIL resistance. With some cell lines, it was indicated that simultaneous EGFR inhibition might sensitize the cells to TRAIL-induced apoptosis. It was also reported that continuous exposure of tumor cells to ENbs is more effective than a single high dose (109).

hMSCs were engineered to express an scFv Ab against EGFRvIII on the cell surface (Table 2). Engineered MSCs showed enhanced binding to U87-EGFRvIII cells *in vitro* and an increased retention in U87-EGFRvIII expressing tumors *in vivo* (110). Down regulation of pAkt was also observed. The growth of U87-EGRFvIII xenografts was inhibited, and survival was significantly improved after *in vivo* treatment with scFvEGRFvIII hMSCs. An additional injection of engineered hMSCs further prolonged the survival. Adding an additional therapeutic gene to these SCs may boost their therapeutic potential even more. The use of GM MSCs with scFv to target tumor-specific antigens, such as EGFRvIII, might achieve stem cell accumulation at the tumor site and prolong therapeutic effect (112).

The presented potential therapies were all performed using intracranial or intravenous injection of SCs, but the first method is invasive and not optimal for repeated administrations. The second method does not deliver the largest number of cells to the brain and can lead to off-target effects although intravenously injected SCs have the potential to cross the BBB and localize to tumors. Intranasal delivery is showing promise in overcoming this challenge. Studies have shown that the intranasal delivery of MSCs or NSCs modified for drug delivery can prolong the survival of glioma animal models (113, 114).

## T-Cell Therapy

In recent years, adoptive T-cell transfer therapy was developed, where tumor-specific T cells are rapidly expanded *ex vivo* and transferred to patients. T cells used in therapy can also be modified to increase their specificity and survival or become resistant to immune evasion mechanisms. Activated T cells (ATC) can cross the BBB irrespective of their antigen specificity, so they are suitable for glioma therapies (115, 116). A chimeric antigen receptor (CAR) can be inserted that encodes Ab fragments specific for tumor-associated antigens. CARs provide T-cell activation regardless of MHC-restricted presentation (117). Potential glioma-specific antigens currently targeted by CAR-T are HER2 (118), EGFRvIII (119–121), EphA2 (122), and IL13Rα2 (123, 124).

A promising use of this technique in glioma therapy is arming anti-CD3-activated T cells with bsAbs that target the T-cell receptor and the tumor-associated antigen and can redirect the non-MHC-restricted cytotoxicity to ATC to lyse tumors. Good targets for this treatment are antigens expressed on glioma stem cells (GSCs). It was reported that arming ATC with either HER2 or EGFR bsAb converts ATC into a specific cytotoxic T cell (125). A recombinant bsAb against the epitopes CD133 and CD3 was developed and locally applied together with autologous CD8^+^ cells. The bsAb redirected polyclonal T cells to CD133^+^ GSCs, where it induced their targeted lysis and prevented the outgrowth of glioblastoma xenografts (126).

## Clinical Trials Overview

In May 2017, over 70 active clinical trials (including pilot studies) addressing the use of Abs in gliomas were registered at clinicaltrials.gov (Table S1 in Supplementary Material). The roles of Abs in these studies are various and include Abs used as agonistic or antagonistic drugs individually or in combination with other Abs, other biologicals, chemotherapeutics, radiotherapeutics, or surgery. Moreover, the combinatorial use of Abs makes them an invaluable tool (e.g., vehicle) in Ab-drug conjugates, Ab-radiodrug conjugates or (with tremendous gain of popularity; Table 3) a part of a molecular construct expressed on the cell surface (CAR on T cells) to bring the drug/toxin, radiodrug or a therapeutic cell (a payload) to its specific antigen target in glioma.

**Table 3:** Overview of the current phase III clinical trials in Ab-based drugs.

	Drug	Target antigen	Ab Type	Phase	Cancer type	Sponsor
1	Bevacizumab (with or w/o Vorinostat, Temozolomide, radiation)	VEGF-A	humanized monoclonal Ab	Ph II, Ph III	High-Grade Glioma	National Cancer Institute (NCI), USA
2	Bevacizumab (with or w/o Lomustine)	VEGF-A	humanized monoclonal Ab	Ph III	Recurrent glioblastoma	European Organisation for Research and Treatment of Cancer—EORTC
3	Bevacizumab (combined with or w/o Temozolomide and radiation)	VEGF-A	Humanized monoclonal Ab	Ph III	Glioblastoma	National Cancer Institute (NCI), USA
4	Nivolumab (with or w/o Bevacizumab and Ipilimumab)	PD-1 VEGF-A CTLA-4	Human monoclonal Ab Humanized monoclonal Ab Human monoclonal Ab	Ph III	Recurrent Glioblastoma	Bristol-Myers Squibb
5	Nivolumab (with or w/o Temozolomide, Radiation)	PD-1	Human monoclonal Ab	Ph III	Glioblastoma	Bristol-Myers Squibb

*Currently, the most commonly targeted antigen in glioma by Ab-based drugs is VEGF-A, followed by programmed cell death protein 1 (PD-1) and cytotoxic T-lymphocyte-associated antigen 4 (CTLA-4). All biological treatments include chemo- and/or radio-therapy or the use of other biologicals*.

The predominant therapeutic Ab-based drugs in these trials are the humanized blocking Abs anti-VEGF-A (Bevacizumab) and human anti-PD1 Ab (Nivolumab). As part of more complex therapy regimens, humanized anti-VEGF-A Abs and human anti-PD1 Abs are also among the five current phase III clinical trials (Table 3). Both bevacizumab (127–129) and nivolumab (130–133) have been a part of clinical trials of glioma for some time alone or combined with other treatment types. Bevacizumab is currently FDA approved for the treatment of glioblastoma that recur after treatment. However, it may be used in the off-label setting if the treating physician prescribes this treatment. “Off-label” indicates the use of an approved treatment for any purpose other than that described in the treatment’s FDA-approved labeling (American Brain Tumor Association).

Fragment Ab-based drugs tested in current clinical trials are used as a vehicle in ADC (D2C7-immunotoxin) (42, 134); or, in most clinical trials, as a part of therapeutic CAR-T cells described in the previous sections, or CAR-pNK cells in one case. The CARs in current studies are led by scFv against five antigens (HER-2, EGFRvIII, MUC-1, IL13Rα2, and EphA2), specific for antigens expressed on glioma cells and/or other solid tumors. As opposed to the biological or combined drugs used in Table 3 and Table S1 in Supplementary Material, in the case of CAR-T (Table 4), we are dealing with cellular therapeutics and, thus, potentially an additional problem in reaching the target by breaching the BBB. However, only one of the current clinical trials on CAR-T cells uses an intratumoral or intracavitary or intraventricular administration of CAR-T cells (Table 4). This implies that the BBB (impaired in brain malignancies) can be adequately breached and that therapeutic cells migrate toward and act against a specific antigen-labeled tumor cell (135, 136).

**Table 4:** Recent chimeric antigen receptor-T cell (CAR-T)-based clinical trials in glioma.

	Biological/drug	Target	Ab type	Clinical Trial Phase	Cancer Type	Sponsor
1	HER2-specific T cells	HER2	scFv	Ph I	Glioblastoma	Nabil Ahmed, Baylor College of Medicine, USA
2	Genetically modified HER.CAR CMV-specific CTLs	HER2	scFv	Ph I	Glioblastoma	Nabil Ahmed, Baylor College of Medicine, USA
3	Anti-EphA2 CAR-T	EphA2	scFv	Ph I, Ph II	Malignant glioma	Fuda Cancer Hospital, Guangzhou, China
4	Anti-EGFRvIII CAR-T (with Aldesleukin, Fludarabine, Cyclophosphamide)	EGFRvIII	scFv	Ph I, Ph II	Malignant glioma	National Cancer Institute (NCI), USA
5	Anti-MUC1 CAR-T cells	MUC-1	scFv	Ph I, Ph II	MUC-1 positive solid tumors, glioma	PersonGen BioTherapeutics (Suzhou) Co., Ltd., China
6	IL13Rα2-specific, hinge-optimized, 41BB-costimulatory CAR/truncated CD19-expressing Autologous T lymphocytes	Interleukin-13 receptor alpha 2 (IL13Rα2)	scFv	Ph I	Malignant glioma	City of Hope Medical Center, USA
7	Anti-MUC1 CAR-pNK cells^a^	MUC-1	scFv	Ph I, Ph II	MUC-1 positive solid relapsed or refractory tumor, glioma	PersonGen BioTherapeutics (Suzhou) Co., Ltd., China
8	Anti-HER2 CAR-T	HER-2	scFv	Ph I, Ph II	HER2 Positive Cancer, glioma	Zhi Yang, Southwest Hospital, China
9	EGFRvIII CAR T cells	EGFRvIII	scFv	Ph I	Glioblastoma	Gary Archer Ph.D., Duke University Medical Center, USA
10	CMV-specific cytotoxic T lymphocytes expressing CAR targeting HER2 (HERT-GBM)	HER-2	scFv	Ph I	Glioblastoma	Nabil Ahmed, Baylor College of Medicine, USA
11	HER2-specific T cells (iCAR)	HER-2	scFv	Ph I	Glioblastoma	Nabil Ahmed, Baylor College of Medicine, USA

*Currently, the most commonly targeted antigens in glioma by CAR-T based cell therapy is HER-2, followed by EGFRvIII, MUC1, EphA2, and IL13Rα2*.

*^a^These studies also include anti-MUC1 CAR-pNK cells, where NK cells are used in place of T cells. Most of the trials use a single type of therapy, without preconditioning*.

## Discussion

Targeting brain tumors and other brain diseases represents a major issue because of the inaccessibility of brain tissue for therapeutics, especially biologics. The aim of therapy is to achieve specific targeting to brain tissue and further on to tumor tissue. Although potential glioma-specific antigens have been identified (118–124, 137–142), the major obstacle still resides in the (in)ability for the specific passage of therapeutics through the BBB to reach tumor tissue in adequate concentrations. In the past few years, many different mechanisms for reaching brain pathologies have been investigated. A Trojan horse method seems especially attractive where Abs and CPPs represent the key players. In the role of a Trojan horse, Abs have already successfully mediated the passage of liposomes containing chemotherapeutics (58), other therapeutic Abs (37), and nanoparticles carrying therapeutic peptides (65). Based on targeting the TfR receptor, it has been shown that Ab valency (32) and affinity (30, 31) are crucial for efficient RMT, and caution must be taken when designing new Abs to mediate RMT. Anti-TfR Abs provide an important insight into how important tuning the interaction and mechanism of interaction can be for the efficient passage through the BBB. When we find an appropriate target and raise an Ab against it, we must evaluate the most appropriate avidity of the therapeutic Ab that would allow the most efficient transcytosis, without redirecting it to the lysosomal pathway. This process is most likely dependent on the target receptor and epitope. To maximize uptake and exposure of a therapeutic Ab, a therapeutic dose must be selected. The saturation concentration of the receptor and decrease in the Ab concentration over time must be considered. Another key player to mediate RMT are CPPs, and they have already successfully mediated the delivery of therapeutic Abs (44), liposomes containing chemotherapeutics (47) and nanoparticles (46). The design of Abs conjugated to CPPs is relatively simple and flexible due to their small size as we have shown previously (18). CPPs present a prospective method to increase the brain uptake of therapeutic Abs. Regarding therapeutic strategies, liposomes and nanoparticles have gained interest and have shown promise as carriers for therapeutics. bsAbs, combining the role of a Trojan horse and a therapeutic agent, have been investigated only for targeting Alzheimer’s disease (30, 68), and their promise as a therapeutic agent for glioblastoma remains to be seen. bsAbs have already been investigated for targeting glioblastoma in the role of mediating a T-cell response (143) and targeting two antigens simultaneously (71, 144). We have not discussed the role of nanobodies for targeting brain diseases. They appear promising since they possess an advantage of high stability, solubility, and small size, providing better tissue penetration, as well as low immunogenicity. A small molecular size and high isoelectric point (pI) have been shown to influence their passive passage through the BBB, possibly *via* ATM (145). However, their concentration in brain tissue remains low (146), and further investigation regarding their passage through the BBB and modifications is needed to evaluate their full therapeutic potential for targeting brain tumors. Although there have been some advances in the discovery of mechanisms for the passage through the BBB, most *in vivo* experiments on brain tumor models still do not investigate the passage and needed concentration for the efficiency of Ab-based therapeutics in orthotopic brain tumor models (Table 1). Many evaluations of potential therapeutics targeting brain tumors circumvent this obstacle by using CED, i.c. administration or non-orthotopic models. Many different Ab-based therapeutic strategies are currently known that present promising future therapies against glioblastoma. However, deeper knowledge regarding the passage through the BBB, identification of new target receptors, Trojan horse agents, and more research in the field of novel therapeutics design and combinational therapy will provide the tools needed for more efficient and safer treatment of brain tumors. The major pitfall resides in the evaluation of the bioavailability of Abs needed to exert their therapeutic potential in the brain. Only few studies have quantitatively assessed the Abs’ capacity to pass the BBB and remain in the brain (26, 147, 148). Also the same methods must be used for evaluation of the capacity to allow comparison among them. Different Abs have different biochemical characteristics, such as amino-acid sequence, isoelectric point, and degree of hydrophobicity. These characteristics can affect the Abs’ physiological properties, including capability to cross the BBB and remain in the brain parenchyma (26). Therefore, we must be careful when comparing capacities of different Abs (e.g., polyclonal Abs to monoclonal Abs) to cross the BBB. Another issue is to extrapolate the findings found in mouse models to humans. Therefore, more studies quantitatively evaluating the capacity of different Ab-based therapies must be performed with the same methods for the evaluation of these properties and allowing comparison between them.

Using SCs for the delivery of therapeutic proteins, including Abs to tumors, seems to be a promising mode of anti-glioma therapy. The main advantages are the ability to cross the BBB and tumor tropic properties, while the largest disadvantages presently are the lack of experience with this sort of therapy and its potential side effects. The results of the first in-human study [NCT01172964 (149)] provided the base for future SC-based clinical trials for patients with brain tumors (primary or metastatic). The NSC cell line used in the study could be further used for the delivery of other anti-tumor drugs, such as Abs. The principle can also be used for other SCs in clinical trials. Main issues that still need to be resolved are the SC lineage and source, immunogenicity, and route of administration. The mechanisms underlying tumor tropism, crossing the BBB and other therapeutic advantages of SCs need to be studied further. Currently, only a few preclinical studies use stem cells as delivery vehicles for Abs or Ab fragments against brain tumors, but they show the potential for the use of Ab-expressing SCs in future clinical studies. It is important that an Ab specific for glioma cells is used that has an adequate therapeutic effect. This calls for meta studies to identify and functionalize reliable glioma-specific markers that could be used as targets to identify and remove these cells.

Adoptive T-cell transfer represents a promising technique in future anti-glioma therapy, especially the use of CARs, which encode scFv Abs specific for tumor-associated antigens fused with endo- and transdomains. However, there are still many challenges to overcome before routine clinical use. Some of these include the loss of antigen in recurring tumors and safety concerns if the antigen is also recognized at low levels in healthy cells.

The number and variety of current clinical trials (Tables 3 and 4; Table S1 in Supplementary Material) show a strong interest in Abs as therapeutic tools. As therapeutic tools Abs can be used either as an active component, vehicle or else. The form of Abs in pharmaceutical formulation can include Abs either as a whole molecule or fragments and can be used either individually or combined with another type of treatment. The frequency of certain therapeutics being used in clinical trials individually or in combination narrows down the current antigens of interest for the future development of Ab-based drugs. Certain drugs being used in other tumors are also being tested in gliomas. The experience with one of the most common Ab-based drugs being used in glioma in clinical trials in the past years, an anti-angiogenic drug, showed that its application changed the tumor phenotype by increasing hypoxia and leading to a metabolic switch toward glycolysis (128, 142). This metabolic switch, in turn, led to increased cell invasion in glioblastoma (150). The metabolic adaptability of GBM cells highlights the difficulty of targeting one specific metabolic pathway for effective therapeutic intervention (151). Thus, by suppressing one specific metabolic pathway, other fronts emerge that we may not be able to anticipate. Currently, the way this is being handled is by combining anti-angiogenic treatment with others (Table 3). Also, a reliable tumor cell marker must be most thoroughly investigated and functionalized preclinically prior to defining it as an adequate drug target.

Therefore, the strongest issues noted here that need to be addressed in the future remain (i) the ability of the Ab-based drug to pass the BBB and reach therapeutic concentrations *in situ*, (ii) functional, fully characterized tumor-specific antigens that would limit the delivery or action of the Ab to tumor cells only and minimize the (cytotoxic, invasive, or else) side effects, and (iii) the immunogenicity of biological and cell-based therapies.

## Author Contributions

RR, NN, VČŠ, and UR contributed to the conception and design of this work, drafted the work, approved of the final version to be published and agreed to be accountable for all aspects of the work in ensuring that questions related to the accuracy or integrity of any part of the work are appropriately investigated and resolved.

## Conflict of Interest Statement

The authors declare that the research was conducted in the absence of any commercial or financial relationships that could be construed as a potential conflict of interest.

## Supplementary Material

Table S1 Clinical Trials Ph I, II.
