# Supplementary material for: Targeting Malignant Brain Tumors with Antibodies
Source: Front Immunol. 2017 Sep 25;8:1181. doi: 10.3389/fimmu.2017.01181 (PMC5622144; doi:10.3389/fimmu.2017.01181)
Supplement: Table S1 — Clinical Trials Ph I, II. [file Table_1.docx]

|  | **Drug** | **Target antigen** | **Ab Type** | **Phase** | **Cancer type** | **Sponsor** |
| --- | --- | --- | --- | --- | --- | --- |
| 1 | 124I-8H9 | B7-H3 | Murine IgG1 | Ph I | Non-Progressive Diffuse Pontine Gliomas | Memorial Sloane Kettering Cancer Center, USA |
| 2 | Bevacizumab (with or w/o Hypofractionated XRT + Temozolomide) | VEGF-A | humanized monoclonal antibody | Ph II | Recurrent Gliomas | Northwestern University, USA |
| 3 | Pidilizumab | PD-1 immune checkpoint | humanized monoclonal antibody | Ph I, Ph II | Gliomas | Hadassah Medical Organization, Israel |
| 4 | Varlilumab (with or w/o IMA950 Vaccine Plus Poly-ICLC) | CD27 | Agonist human mAb (IgG1k) | Ph I | Low grade glioma | University of California San Francisco, USA |
| 5 | 1. Tremelimumab 2. Durvalumab | 1. CTLA 4 2. CD274 | 1. human monoclonal antibody 2. human (IgG1κ) monoclonal antibody | Ph II | Gliomas | Northwestern University, USA |
| 6 | Bevacizumab (with or w/o ascorbic acid) | VEGF-A | humanized monoclonal antibody | Ph I | High Grade Glioma | University of Nebraska, USA |
| 7 | D2C7- PE38KDEL (ADC) | EGFRwt, EGFRvIII | single-chain disulfide stabilized fragment variable (scdsFv) mAb | Ph I | Glioma | Darell D. Bigner, Duke University Medical Center, USA |
| 8 | Pembrolizumab | programmed cell death 1 (PD-1) receptor | humanized monoclonal antibody | Pilot Study | Glioma | Memorial Sloan Kettering Cancer Center |
| 9 | Nivolumab (with DC vaccine) | PD-1 | Human monoclonal antibody | Ph I | Glioma | Gary Archer, Duke University Medical Center, USA |
| 10 | Pembrolizumab | programmed cell death 1 (PD-1) receptor | humanized monoclonal antibody | N/A | Glioma, Lynch Syndrome,  Mismatch Repair Gene Inactivation,  Recurrent Childhood Brain Neoplasm,  Refractory Brain Neoplasm | National Cancer Institute (NCI), USA |
| 11 | Bevacizumab (with temozolamide) | VEGF-A | humanized monoclonal antibody | Ph II | Central Nervous System Tumors | European Organisation for Research and Treatment of Cancer - EORTC |
| 12 | Cetuximab (with cetuximab-IRDye 800, surgery) | EGFR | Chimeric | Ph I, Ph II | Glioma | Eben Rosenthal, Stanford University, USA |
| 13 | Bevacizumab | VEGF-A | humanized monoclonal antibody | Ph II | Progressive Glioma | Northwestern University, USA |
| 14 | Sym004 | EGFR | A mixture of two recombinant IgG1 against different epitopes of EGFR extracellular domain III | Ph II | Recurrent Glioblastoma | Annick Desjardins, Duke University Medical Center, USA |
| 15 | Bevacizumab (with or w/o Toll-like Receptor Agonist as an Adjuvant to Tumor Associated Antigens (TAA) Mixed With Montanide ISA-51 VG) | VEGF-A | humanized monoclonal antibody | Ph II | Glioma | New York University School of Medicine, USA |
| 16 | Bevacizuma (with or w/o desatinib) | VEGF-A | humanized monoclonal antibody | Ph II | High grade glioma | Alliance for Clinical Trials in Oncology, USA |
| 17 | Nivolumab  (with or w/oHypofractionated Stereotactic Irradiation) | PD-1 | human monoclonal antibody | Ph I | Malignant Glioma | H. Lee Moffitt Cancer Center and Research Institute, USA |
| 18 | 1. Bevacizumab 2. TRC 105 mAb | 1. VEGF-A 2. endoglin | 1. humanized monoclonal antibody 2. chimeric mAb | Ph I, Ph II | Recurrent Adult Brain Neoplasm,  Recurrent glioma | National Cancer Institute (NCI), USA |
| 19 | 177Lu Radiolabeled Monoclonal Antibody HuJ591-GS (177Lu-J591; | prostate specific membrane antigen (PSMA) | Humanized mouse mAb | Pilot study | Non-prostate solid tumors, gliomas | Weill Medical College of Cornell University, USA |
| 20 | Bevacizumab (with or w/o Hypofractionated Stereotactic Radiotherapy) | VEGF-A | humanized monoclonal antibody | Ph I | Glioma | Memorial Sloan Kettering Cancer Center, USA |
| 21 | Cetuximab (with or w/o reirradiation) | EGFR | Chimeric mAb | Ph II | Refractory glioma | John A. Boockvar, Northwell Health, USA |
| 22 | ABT-414 (ABT-806 linked to monomethyl auristatin F; with or w/o temozolamide and Limoustine) | EGFR, EGFRvIII | humanized recombinant IgG1κ | Ph II | Pediatric high grade gliomas | AbbVie |
| 23 | 89Zr-J591 (Zr labeled mAb J591) | prostate specific membrane antigen (PSMA) | Humanized mouse mAb | Pilot study | Glioblastoma | Memorial Sloan Kettering Cancer Center, USA |
| 24 | Pembrolizumab  (with or w/o DNX-2401 oncolytic virus) | PD-1 | Humanized mouse mAb | Ph II | Glioma | DNAtrix, Inc., USA |
| 25 | IMMU-132 (hRS7-SN38 Antibody Drug Conjugate; | TROP-2 antigen | humanized monoclonal antibody | Ph I, Ph II | Epithelial cancer, Glioblastoma | Immunomedics, Inc. |
| 26 | anti-PD-1 antibody (with or w/o INT230-6) | PD-1 | Humanized mouse mAb | Ph I, Ph II | Refractory cancers, glioblastoma | Intensity Therapeutics, Inc., USA |
| 27 | 1. BMS-986016 2. Nivolumab 3. Urelumab | 1. LAG3 2. PD1 3. CD-137 | 1. Human monoclonal Ab 2. Human monoclonal Ab 3. Human monoclonal Ab | Ph I | Recurrent glioblastoma | Sidney Kimmel Comprehensive Cancer Center, USA |
| 28 | Nivolumab  (with or w/o Dendritic Cells Pulsed With Tumor Lysate Antigen Vaccine) | PD-1 | Human monoclonal antibody | Ph II | Recurrent glioblastoma | Jonsson Comprehensive Cancer Center, USA |
| 29 | 1. Cabiralizumab (FPA008) 2. Nivolumab | 1. colony stimulating factor-1 receptor (CSF1R) 2. PD-1 | 1. Humanizedmonoclonal antibody 2. Human monoclonal antibody | Ph I | Selected advanced cancers, malignant glioma | Five Prime Therapeutics, Inc., USA |
| 30 | 1. Nivolumab 2. Varlilumab | 1. PD-1 2. CD-27 | 1. Human monoclonal antibody 2. Human monoclonal antibody | Ph I, Ph II | Advanced Refractory Solid Tumors, glioblastoma (Ph II only) | Celldex Therapeutics, USA |
| 31 | TTAC-0001(Tanibirumab) | VEGFR-2 | Human monoclonal antibody | Ph II | Recurrent glioblastoma | PharmAbcine, Korea |
| 32 | Bevacizumab  (with or w/o radiation) | VEGF-A | humanized monoclonal antibody | Ph II | Recurrent Adult Brain Tumor, glioblastoma | Radiation Therapy Oncology Group |
| 33 | 1. Ipilimumab 2. Nivolumab   (combined with or w/o Temozolamide) | 1. CTLA-4 2. PD-1 | 1. Human monoclonal antibody 2. Human monoclonal antibody | Ph I | Supratentorial Glioblastoma | National Cancer Institute (NCI), USA |
| 34 | Bevacizumab  (TORC1/2 Inhibitor MLN0128) | VEGF-A | humanized monoclonal antibody | Ph I | Solid tumors, glioblastoma | National Cancer Institute (NCI), USA |
| 35 | Bevacizumab (combined with or w/o Cediranib Maleate and Olaparib) | VEGF-A | humanized monoclonal antibody | Ph II | Recurrent glioblastoma | National Cancer Institute (NCI), USA |
| 36 | Bevacizumab (combined with or w/o Temozolamide) | VEGF-A | humanized monoclonal antibody | Ph II | Glioblastoma | Jonsson Comprehensive Cancer Center, USA |
| 37 | Bevacizumab  (combined with or w/o Trebananib) | VEGF-A | humanized monoclonal antibody | Ph II | Glioma | National Cancer Institute (NCI), USA |
| 38 | Basiliximab (combined with or w/o RNA-loaded dendritic cell vaccine) | CD-25 | Chimeric | Ph I | Glioblastoma | John Sampson, Duke University Medical Center, USA |
| 39 | Panitumumab (an Ab component of EGFR(V)-EDV-Dox) | EGFR | bispecific antibodies (BsAb) | Ph I | Glioblastoma | Engeneic Pty Limited, Australia |
| 40 | Bevacizumab (with or w/o Optune(NOVOTTF-100A and Hypofractionated Stereotactic Irradiation) | VEGF-A | humanized monoclonal antibody | Pilot Study | Recurrent Glioblastoma | Department of Radiation Oncology, University of Maryland, USA |
| 41 | Bevacizumab (with or w/o sorafenib tosylate) | VEGF-A | humanized monoclonal antibody | Ph II | Glioblastoma | Alliance for Clinical Trials in Oncology |
| 42 | Bevacizumab (and erlotinib after temozolamide and irradiation)) | VEGF-A | humanized monoclonal antibody | Ph II | Glioblastoma | Jeffrey Raizer, Northwestern University, USA |
| 43 | Bevacizumab (and temozolamide and external beam radiation) | VEGF-A | humanized monoclonal antibody | Ph II | Glioblastoma | Jonsson Comprehensive Cancer Center, USA |
| 44 | Bevacizumab (and HSPPC-96 tumor vaccine) | VEGF-A | humanized monoclonal antibody | Ph II | Glioblastoma | Alliance for Clinical Trials in Oncology |
| 45 | Pembrolizumab (MK-3475); (with or w/o pictilisib –Suppressor of the PI3K/Akt pathways) | PD-1 | humanized monoclonal antibody | Ph I, Ph II | Glioblastoma | Ms. Jann Lee, Medical Research Council |
| 46 | Bevacizumab (and NovoTTF-l00A) | VEGF-A | humanized monoclonal antibody | Ph II | Glioblastoma | Case Comprehensive Cancer Center, USA |
| 47 | Durvalumab (and Hypofractionated stereotactic radiation therapy) | CD274 |  | Ph I, Ph II | Glioblastoma | Institut Claudius Regaud, France |
| 48 | 1. Bevacizumab 2. (with or w/o Durvalumab) | 1. VEGF-A 2. CD274 | 1. humanized monoclonal antibody 2. human monoclonal antibody | Ph II | Glioblastoma | Ludwig Institute for Cancer Research, USA |
| 49 | Nivolumab (and Epacadostat) | PD-1 | Human monoclonal antibody | Ph I, Ph II | Advanced cancers, glioblastoma | Incyte Corporation, USA |
| 50 | Bavituximab (and temozolamide and radiation) | phosphatidylserine | Chimeric | Ph II | Glioblastoma | Elizabeth R. Gerstner, MD, Massachusetts General Hospital, USA |
| 51 | Nivolumab (and Valproate and stereotactic radiosurgery) | PD-1 | Human monoclonal antibody | Ph I | Glioblastoma | Benjamin Purow, MD, University of Virginia |
| 52 | ABT-414 (ABT-806 linked to monomethyl auristatin F; with temozolamide and radiation) | EGFR, EGFRvIII | humanized recombinant IgG1κ | Ph II | Glioblastoma | AbbVie |
| 53 | Basiliximab (with or w/o Unpulsed DCs, Td, Human CMV pp65-LAMP mRNA-pulsed autologous DCs, 111In-labeled DCs, Temozolomide, Saline) | CD-25 | Chimeric | Ph II | Glioblastoma | Gary Archer Ph.D., Duke University Medical Center, USA |
| 54 | Nivolumab  (with or w/o Temozolomide, Radiation, Nivolumab Placebo) | PD-1 | Human monoclonal antibody | Ph II | Glioblastoma | Bristol-Myers Squibb |
| 55 | Rovalpituzumab tesirine (ADC – anti DLL3 conj. to pyrrolobenzodiazepine dimer) | DLL3 | Humanized monoclonal antibody | Ph I, Ph II | Advanced solid tumors, Glioblastoma | Stemcentrx |
